# An Approach to Inferring Transcriptional Regulation Among Genes From Large-Scale Expression Data

**DOI:** 10.1002/cfg.237

**Published:** 2003-02

**Authors:** Javier Herrero, Ramón Díaz-Uriarte, Joaquín Dopazo

**Affiliations:** Unidad de Bioinformática Centro Nacional de Investigaciones Oncológicas (CNIO). Melchor Fernández Almagro 3 Madrid 28029 Spain

## Abstract

The use of DNA microarrays opens up the possibility of measuring the expression levels of thousands of genes simultaneously under different conditions. Time-course experiments allow researchers to study the dynamics of gene interactions. The inference of genetic networks from such measures can give important insights for the understanding of a variety of biological problems. Most of the existing methods for genetic network reconstruction require many experimental data points, or can only be applied to the reconstruction of small subnetworks. Here we present a method that reduces the dimensionality of the dataset and then extracts the significant dynamic correlations among genes. The method requires a number of points achievable in common time-course experiments.


**Comparative and Functional Genomics**
*Comp Funct Genom* 2003; **4**: 148–154.
Published online in Wiley InterScience (www.interscience.wiley.com). **DOI:** 10.1002/cfg.237

**Conference Paper**

### An approach to inferring transcriptional regulation among genes from large-scale expression data

Javier Herrero, Ramón Díaz-Uriarte and Joaquín Dopazo*
*Unidad de Bioinformática, Centro Nacional de Investigaciones Oncológicas (CNIO). Melchor Fernández Almagro 3, 28029 Madrid, Spain*

*Correspondence to: Joaquín Dopazo, Unidad de Bioinformática, Centro Nacional de Investigaciones Oncológicas (CNIO). Melchor Fernández Almagro 3, 28029 Madrid, Spain. E-mail: jdopazo@cnio.es*

Received: 11 July 2002
Accepted: 22 November 2002

### Abstract

**The use of DNA microarrays opens up the possibility of measuring the expression levels of thousands of genes simultaneously under different conditions. Time-course experiments allow researchers to study the dynamics of gene interactions. The inference of genetic networks from such measures can give important insights for the understanding of a variety of biological problems. Most of the existing methods for genetic network reconstruction require many experimental data points, or can only be applied to the reconstruction of small subnetworks. Here we present a method that reduces the dimensionality of the dataset and then extracts the significant dynamic correlations among genes. The method requires a number of points achievable in common time-course experiments.** **Copyright © 2003 John Wiley & Sons, Ltd.**

**Keywords:** **DNA microarrays; gene expression; clustering reverse engineering; genetic networks; time course; SOTA**

### Introduction

Understanding the structure of genetic networks is an essential step towards the comprehension of a variety of problems in biology. Gene interaction networks, even in the simplest organisms, are only starting to be elucidated in detail. The advent of genomic technologies has made available a huge amount of information on gene interactions from large-scale disruption experiments (Fraser *et al.*, 2000; Hughes, 2000; Winzeler *et al.*, 1999), or from genome-wide two-hybrid assays (Ito *et al.*, 2001). This information is far from being detailed, and in many cases accounts only for physical binding of unknown nature (which might even not occur within the cell), positive or negative correlated expression, or direct or indirect interactions among genes. In some cases, the information provided by DNA microarray experiments allows us to explore such interactions from a more causal perspective.

Perturbation of genes, or different treatments, are used to obtain a variety of gene expression profiles reflecting their interactions in pathways. Thus, the comparison of wild-type and mutant profiles allows the identification of downstream genes, whose expression is affected by the specific mutation of a gene (Holstege *et al.*, 1998). Nevertheless, perturbation data cannot distinguish direct from indirect effects (Wagner, 2002). The study of time series opens up the possibility of taking into account the dynamics of the gene interactions in the inference of the network (Li *et al.*, 2002). This inference process, called 'reverse engineering', aims to determine the cause (the network of interactions) from the effect (the observed changes in gene expression levels) (D'haeseleer *et al.*, 2000).

The simplest, pure *in silico,* approach consists of clustering the data and searching for regulatory control elements (e.g. promoters) in all co-expressing genes (Brazma *et al.*, 1998; Tavazoie *et al.*, 1999). Nevertheless, the information provided by these approaches is limited to genes that are co-regulated, rather than which genes are regulating which other genes. In network inference, the aim is to construct a model of the

Copyright © 2003 John Wiley & Sons, Ltd.

interactions between genes. This requires inference of the causal relationships among genes or, in other words, the reverse engineering of the network architecture from the gene expression profiles. Boolean networks (Somogyi and Shiegoski, 1996) or Bayesian networks (Friedman *et al.*, 2000) have been utilized for reverse engineering of gene networks. Other models include linear differential equations (D'haeseleer *et al.*, 1999) and non-linear models (Weaver *et al.*, 1999). Recently, probabilistic graphical models have been implemented in Boolean networks, which allowed the study of features such as mediation, activation and inhibition in more depth, and permitted the construction of significant subnetworks (Pe'er *et al.*, 2001). The number of experimental points necessary for reverse engineering genetic networks depends on the connectivity (number of possible gene interactions) and the method used. Fully connected networks would need up to $2^n$ experimental measures for $n$ genes, which is a completely unrealistic number, but introducing restrictions derived from previous knowledge of the system can drastically decrease the number of points required (D'haeseleer *et al.*, 2000). These restrictions usually limit the possibilities of analysis to very small subnetworks.

Observation of correlation between variables has long been used in biology as a predictor of causal relationships. An obvious advantage of correlation is that the number of experimental points necessary are in the range of $\log(n)$ (D'haeseleer *et al.*, 2000). Time-lagged correlation has been used to study the effect of genes at time $t$ over them at $t + 1$ (Arkin *et al.*, 1997; Li *et al.*, 2002). A high correlation (positive or negative) between gene A and gene B at $t + 1$ can be caused by gene A regulating gene B (directly, or through some intermediates) or by accident. Correlation itself is only an indication, but not a proof, of causal relationship. This indication, which can be taken as a hypothesis, must be tested by other means. Recently, bootstrap resampling (Efron and Tibshirani, 1991) has been applied to check the statistical reliability of gene networks obtained from means of time-lagged partial correlations (Toh and Horimoto, 2002). Another important issue is dimensionality reduction (Mjolsness *et al.*, 2000; Toh and Horimoto, 2002). Gene expression profiles over time are highly redundant data because many profiles are virtually identical. Treating them as independent pieces of information will produce a vast amount of virtually identical solutions for any method. We propose here a novel approach that allows the inference of a network of gene interactions at the transcriptional level from time-lagged correlations, and check its reliability by means of a permutation test that takes into account the multiple testing nature of the results. The accuracy of the network is checked by the agreement of the results with available biological data.

### Methods

The data consist of time-course experiments. A first step involves the exclusion of genes with flat profiles, which would produce artifactual positive correlations. Then, the data are clustered using SOTA (Herrero *et al.*, 2001) to obtain a non-redundant dataset of gene expression profiles. Finally, the time-lagged correlation matrix is obtained. If SOTA clustering leads to $n$ clusters, $n*(n - 1)$ coefficients have to be examined. Only pairs of gene clusters with a significant coefficient of correlation will be taken into account as part of the network. Since we are examining thousands of correlation coefficients, we cannot simply use the individual $p$ value of each correlation coefficient to judge the significance of the correlation; in our case, each correlation coefficient is used to examine the null hypothesis of no correlation between the expression of gene A at time $t$ and gene B at time $t - 1$. Thus, we are carrying out thousands of hypothesis tests simultaneously, one corresponding to each correlation coefficient. If we were to consider each of the correlation coefficients with a $p$ value smaller than, say, 0.05, as significant, the Type I family-wise (i.e. over the family of thousands of tests) error rate would be much larger than 0.05 (the Type I error rate is the probability of rejecting the null hypothesis when the null is in fact true). In other words, we would end up with an excessive number of false rejections; thus the need to account for multiple testing.

Most multiple testing procedures are designed to directly control the Type I family-wise error rate (FWER; Westfall & Young, 1993). These methods, however, can be unnecessarily conservative, leading to a lack of power to detect false hypotheses. In this paper, to obtain the significant between gene correlations, we have chosen

Copyright © 2003 John Wiley & Sons, Ltd. 

instead the approach of controlling the false discovery rate (FDR). With the FDR approach we control the expected number of false rejections among the rejected hypotheses (for a simple explanation, see Benjamini *et al.*, 1999; also Dudoit *et al.*, 2002). There are different types of FDR-controlling procedures; the one we use here is that of Benjamini and Yekutieli (2001), which allows for strong control (i.e. control under any combination of true and false hypotheses) of the FDR under arbitrary dependence structures. In this paper, we have used an FDR-adjusted $p$ value of $< 0.05$ as a heuristic rule for selecting relevant correlations. The unadjusted $p$ values were obtained with a permutation test with 1 200 000 random permutations, where the vectors of observations are permuted independently (see e.g. Westfall and Young, 1993, p. 194). Thus, we are resampling under the complete null hypothesis of independence (i.e. that all variables are independent). By using resampling when obtaining the $p$ value, we are accounting for possible non-normality in the data.

A pair of gene clusters were considered to be part of the network if the correlation between them was higher than 0.7, or lower than −0.7, with an adjusted $p$ value $< 0.05$, as explained above. To check if the network we reconstructed had any biological meaning we studied the distribution of gene ontology (GO) terms (Ashburner *et al.*, 2000) among the connected genes and the unconnected genes. Clusters of genes in the network are regarded as connected when they display three or more connections to other gene clusters. We compared the frequency of GO terms from the categories corresponding to molecular function and biological process (Ashburner *et al.*, 2000) between connected and unconnected genes. We used Fisher's exact test: for each GO term we can build a $2 \times 2$ contingency table of type of gene (connected vs. unconnected) by presence/absence of a GO term. Here again, we faced the problem of multiple testing, because the comparison was done for each of several GO terms (from 20 to 58). To account for multiple testing we adjusted the $p$ value from Fisher's exact test using the minP method of Westfall and Young (1993, algorithm 2.8, p. 66; see also Dudoit *et al.*, 2002), based on 10 000 random permutations of the columns of data. The minP method allows for strong control of the FWER.

### Results and discussion

We have used the expression profile of 6178 yeast genes across complete cell cycles of synchronized cells (Eisen *et al.*, 1998). We chose an individual time series corresponding to 18 time points of alpha factor-arrested cells and removed all the genes whose expression profiles along all the points had a root mean square lower than 0.3. Then, the remaining 1453 gene expression profiles were clustered using SOTA (Herrero *et al.*, 2001) with a threshold of 0.435, corresponding to a 90% probability of having true positives in the cluster (for details, see Herrero *et al.*, 2001). This operation removed virtually all the redundancies in the dataset, which was reduced to 439 non-redundant average profiles. These average profiles were obtained by SOTA from the genes contained in each cluster. On average, there are 3.3 genes/cluster, although there are 122 singletons, and clusters with as many as 18 genes. We obtained the matrix of time-lagged correlation for all these profiles. Then, for the 439 profiles we had to examine $439*438 = 192\,282$ coefficients.

After application of the procedure for selecting relevant correlations with an FDR-adjusted $p$ value $< 0.05$ we found a total of 381 significant correlations. Figure 1 shows the network obtained. The bottom of the picture represents the significant positive and negative correlations obtained after the application of the method. The connectivity of the network follows the power law typical of scale-free networks (Barabasi and Albert, 1999), with a $\gamma$ parameter of 1.36, in the range of those that describe this type of networks. This type of connectivity, which makes biological networks robust against perturbations (Wagner, 2000), indicates that, at the connectivity level, the network we found has a biological meaning. The distribution of GO terms (Figure 2) shows that, in the biological process category, the terms 'cell–cell fusion' and 'cell cycle' are significantly overrepresented in the connected genes. On the contrary, 'metabolism' is a significantly underrepresented term. These results are in agreement with what one would expect from such an experiment. Metabolic processes will occur at different points during the cell cycle, and with a complex dynamic that is very difficult to find by studying time-lagged correlation across the whole cell cycle. On the other hand, it is known that processes related to 'cell–cell fusion' and 'cell

Copyright © 2003 John Wiley & Sons, Ltd.

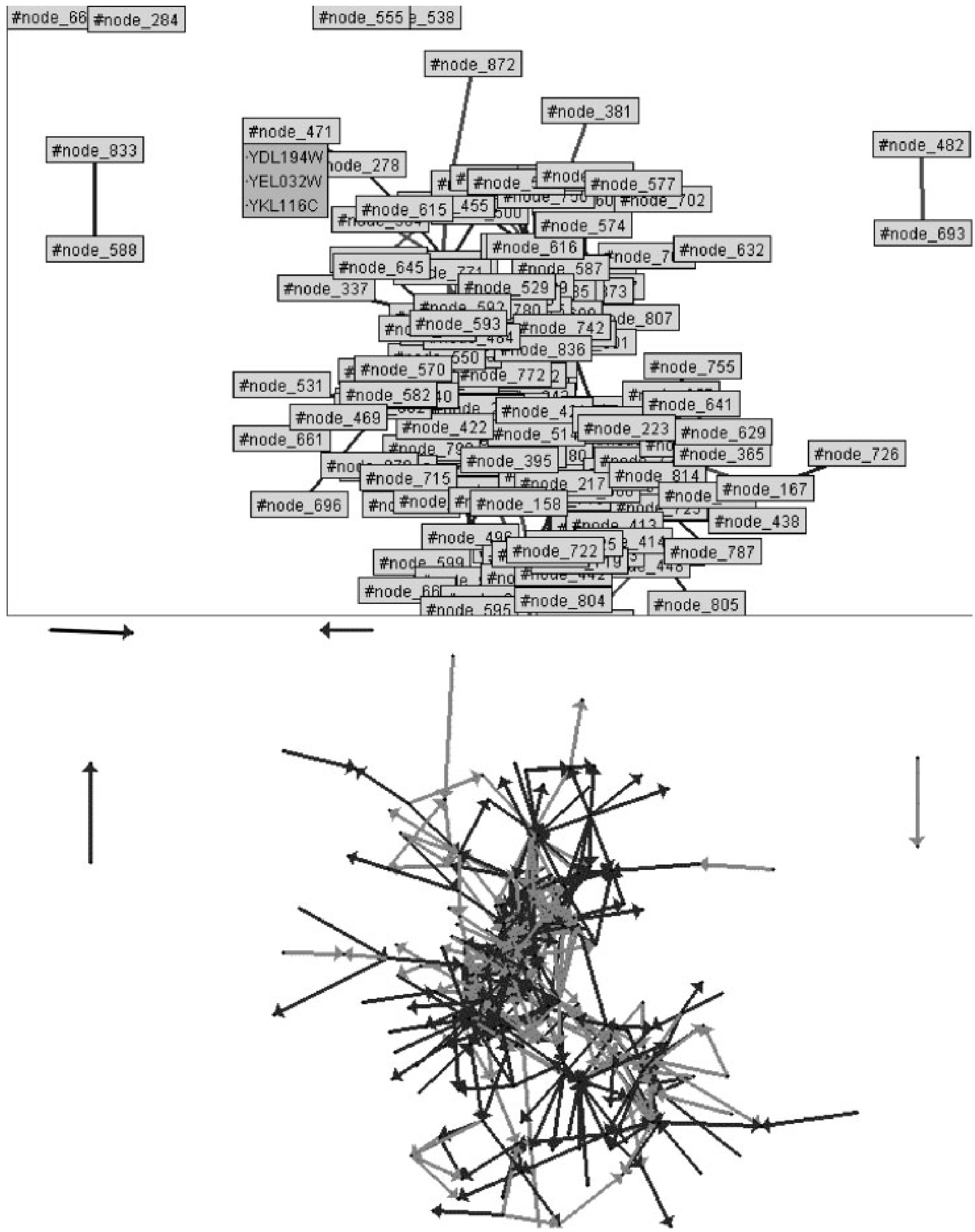

**Figure 1.** Genetic network of transcriptional interactions obtained as significant time-lagged correlations (see text). (Upper panel) Nodes are clusters of co-expressing genes obtained upon the application of SOTA, e.g. #node 471 contains the genes YDL194W, YEL032W and YKL116C. (Lower panel) Schematic representation of the interactions between clusters of co-expressing genes. Black arrows indicate positive correlations and grey arrows represent negative correlations

Copyright © 2003 John Wiley & Sons, Ltd. 

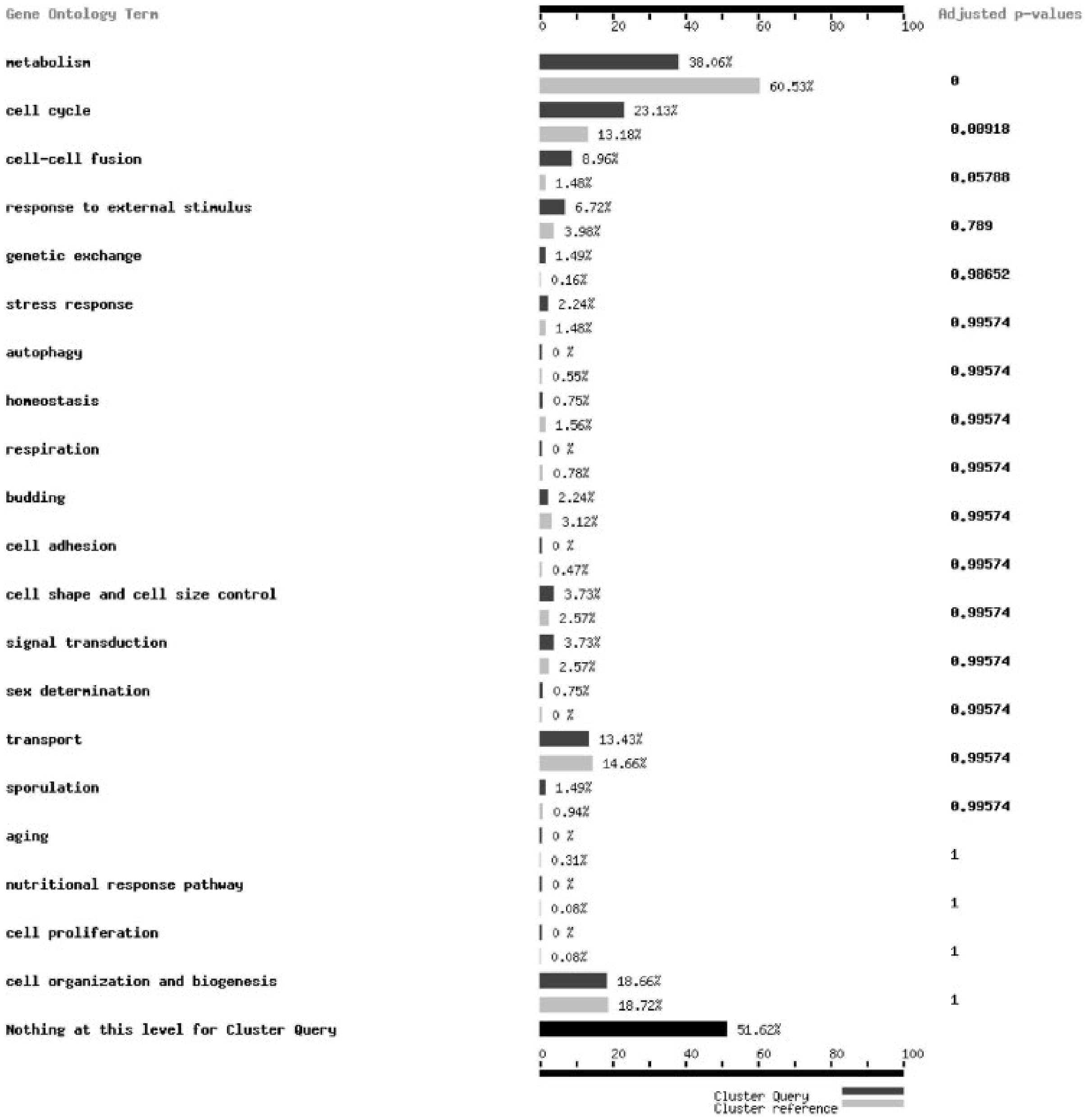

**Figure 2.** The differential distribution of GO terms for biological function between highly connected genes and unconnected genes. See text for an explanation on adjusted $p$ values cycle' are coupled to events of the cell cycle and thus these can be detected through the time-lagged correlations. In fact, when the most connected genes were analysed, many of them turned out to be transcription factors or genes with a DNA binding domain (Table 1). Interestingly, a few of them are genes of unknown function, and our results strongly suggest that these might participate in some regulatory function in cell cycle-related processes. The complete results are available at: **http://bioinfo.cnio.es/data/CFG02**.

Here we have reported a method that combines initial reduction of the dimensionality of the data set with the application of a test that takes into account the multiple testing nature of the simultaneous study of thousands of correlation values. The

Copyright © 2003 John Wiley & Sons, Ltd. 

**Table 1.** Transcription factors or genes that presumably can interact with (activate, or repress) other genes, present among the genes showing a highest degree of interaction in the network

| Systematic name | Standard name | Total connections | Molecular function | Biological process | Description |
|---|---|---|---|---|---|
| YNL068C | FKH2 | 17 | Transcription factor | $G_2$-specific transcription in mitotic cell cycle<br>Pseudohyphal growth<br>Regulation of cell cycle | Fork Head homologue two |
| YPR104C | FHL1 | 14 | Transcription factor | rRNA processing<br>Transcription, from PolIII promoter | Putative transcriptional regulator of rRNA-processing genes |
| YOR372C | NDD1 | 14 | Transcriptional activator | $G_2$/M-specific transcription in mitotic cell cycle | Nuclear Division Defective 1 |
| YBR009C | HHF1 | 9 | DNA binding | Chromatin assembly/disassembly | Histone H4 (HHF1 and HHF2 code for identical proteins) |
| YLL008W | DRS1 | 9 | ATP-dependent RNA helicase | 35S primary transcript processing<br>Ribosomal large subunit assembly and maintenance | Ribosome assembly |
| YML065W | ORC1 | 9 | DNA replication origin binding adenosinetriphosphatase | DNA replication initiation<br>Chromatin silencing at HML and HMR (*sensu Saccharomyces*)<br>Pre-replicative complex formation and maintenance | Binds to origins of replication and thereby directs DNA replication and is also involved in transcriptional silencing |
| YGR109C | CLB6 | 9 | Cyclin-dependent protein kinase, regulator | $G_1$/S transition of mitotic cell cycle<br>Premeiotic DNA synthesis<br>Regulation of CDK activity | Role in DNA replication during S phase |
| YCL024W | KCC4 | 8 | Protein kinase transcription factor | Axial budding<br>Bud growth<br>Protein amino acid phosphorylation<br>Septin assembly and septum formation<br>Septin checkpoint | Involved in septin organization |
| YHR084W | STE12 | 8 | Transcription factor | Invasive growth<br>Pheromone induction of gene expression from Pol II promoter<br>Pseudohyphal growth | Involved in pheromone and pseudohyphal growth signal transduction pathways |

Copyright © 2003 John Wiley & Sons, Ltd.

method is not demanding on data points and can reconstruct large-scale networks from a number of experimental points in the range of realistic experiments. We have shown that the networks recovered in this way are supported by existing gene function annotation. The problem of inferring genetic networks is far from being solved, but approaches like the one reported here constitute robust and operative tools that provide realistic results on the dynamics of gene interactions.

### References

Arkin A, Shen P, Ross J. 1997. A test case of correlation metric construction of a reaction pathway from measurements. *Science* **277**: 1275–1279.

Ashburner M, Ball CA, Blake A, *et al*. 2000. Gene Ontology: tool for the unification of biology. *Nature Genet* **25**: 25–29.

Barabasi AL, Albert R. 1999. Emergence of scaling in random networks. *Science* **286**: 509–512.

Benjamini Y, Drai D, Elmer G, Kafkafi N, Golani I. 1999. Controlling the false discovery rate in behavior genetics research (available from: **http://www.math.tau.ac.il/~ybenja/**).

Benjamini Y, Yekutieli D. 2001. The control of the false discovery rate in multiple testing under dependency. *Ann Statist* **29**(4): 1165–1188.

Brazma A, Jonassen I, Vilo J, Ukkonen E. 1998. Predicting gene regulatory elements *in silico* on a genomic scale. *Genome Res* **8**: 1202–1215.

D'haeseleer P, Liang S, Somogyi R. 2000. Genetic network inference: from co-expression clustering to reverse engineering. *Bioinformatics* **16**: 707–726.

D'haeseleer P, Wen X, Furhman S, Somogyi R. 1999. Linear modeling of mRNA expression levels during CNS development and injury. *Pac Symp Biocomput* **4**: 41–52.

Dudoit S, Shaffer JP, Boldrick JC. 2002. Multiple hypothesis testing in microarray experiments. Technical Report No. 110, Division of Biostatistics, University of California at Berkeley, CA.

Efron B, Tibsirani R. 1991. Statistical data analysis in the computer age. *Science* **253**: 390–395.

Eisen M, Spellman PL, Brown PO, Botstein D. 1998. Cluster analysis and display of genome-wide expression patterns. *Proc Natl Acad Sci USA* **95**: 14 863–14 868.

Fraser AG, Kamath RS, Zipperlen P, *et al*. 2000. Functional genomic analysis of *C. elegans* chromosome I by systematic RNA interference. *Nature* **408**: 325–330.

Friedman N, Linial M, Nachman I, Pe'er D. 2000. Using Bayesian networks to analyze expression data. *J Comput Biol* **7**: 601–620.

Herrero J, Valencia A, Dopazo J. 2001. A hierarchical unsupervised growing neural network for clustering gene expression patterns. *Bioinformatics* **17**: 126–136.

Holstege FC, Jennings EG, Wyrick JJ, *et al*. 1998. Dissecting the regulatory circuitry of a eukaryotic genome. *Cell* **95**: 717–728.

Hughes TR, Marton MJ, Jones AR, *et al*. 2000. Functional discovery via a compendium of expression profiles. *Cell* **102**: 109–126.

Ito T, Chiba T, Ozawa R, *et al*. 2001. A comprehensive two-hybrid analysis to explore the yeast protein interactome. *Proc Natl Acad Sci USA* **98**: 4569–4574.

Li H, Luan Y, Hong F, Li Y. 2002. Statistical methods for the analysis of time course gene expression data. *Front Biosci* **7**: 90–98.

Mjolsness E, Mann T, Castaño R, Wold B. 2000. From co-expression to co-regulation: an approach to inferring transcriptional regulation among gene classes from large-scale expression data. In *Advances in Neural Information Processing Systems, vol 12*, Solla SA, Leen TK, Muller KR (eds). MIT Press: Cambridge, MA, USA. 928–934.

Pe'er D, Regev A, Elidan G, Friedman N. 2001. Inferring subnetworks from perturbed expression profiles. *Bioinformatics* **17**(suppl 1): S215–S224.

Somogyi R, Shiegoski CA. 1996. Modelling the complexity of genetic networks: understanding multigene and pleiotropic regulation. *Complexity* **1**: 45–63.

Tavazoie S, Hughes JD, Campbell MJ, Cho RJ, Church GM. 1999. Systematic determination of genetic network architecture. *Nature Genet* **22**: 281–285.

Toh H, Horimoto K. 2002. Inference of a genetic network by a combined approach of cluster analysis and graphical Gaussian modelling. *Bioinformatics* **18**: 287–297.

Wagner A. 2000. Robustness against mutations in genetic networks of yeast. *Nature Genet* **24**: 355–361.

Wagner A. 2002. Estimating coarse gene network structure from large-scale gene perturbation data. *Genome Res* **12**: 309–315.

Weaver C, Workman C, Stormo G. 1999. Modelling regulatory networks with weight matrices. *Pac Symp Biocomput* **4**: 112–123.

Westfall PH, Young SS. 1993. *Resampling-based Multiple Testing*. Wiley: New York.

Winzeler EA, Shoemaker DD, Astromoff A, *et al*. 1999. Functional characterization of the *S. cerevisiae* genome by gene deletion and parallel analysis. *Science* **285**: 901–906.

Copyright © 2003 John Wiley & Sons, Ltd. 
